# American Text Book of Operative Dentistry

**Published:** 1912-02-15

**Authors:** 


AMERICAN TEXT BOOK OF OPERATIVE DENTISTRY. The fourth edition of the work has been thoroughly revised and much new material added to its pages. We cannot here make a detailed review of its contents, as our time and space would not permit a sufficiently close reading of such a large work to do this conscientiously. We are constrained to remark in passing that the book of over 900 pages might just as well have been 231 pages less bulky by leaving out the work on orthodontia, which we do not believe has a proper place in a work on operative dentistry. As nearly as we can determine the excellent book of Dr. Angle on Orthodontia has been wholly incorporated. A similar innovation was first incorporated in Johnsons Text Book on Operative Dentistry. It is in our judgment a mistake to make encyclopedias of our text books especially for students use, and we doubt whether practitioners will buy such large works as readily as they would a smaller volume which represented the best thought of a single individual. The general plan of the work has not been materially changed, but much new material has been added and almost every subject has undergone more or less revision; this is necessary because of the rapid and somewhat radical changes that have taken place in operative procedures during the past five years. The chapter on Plastics and Combination Fillings by Professor M. L. Ward is new and gives an up-to-date scientific treatment of amalgam, cement and other filling materials and their proper manipulations. The results of modern research are compactly stated and valuable technical suggestions given for using these materials alone or in combinations. If all our operative procedures were adjusted to this kind of work there would be a more uniform and safer basis of practice. The work of Black and Noyes on defective tooth tissues with reference to cavity preparation is an excellent example of what a scientific knowledge of conditions will do to settle methods of technical procedure. The cemented inlay receives good treatment at the hands of Dr. Capon, although his cavity preparation is not as thoroughly done as is his other technical treatment. He gives a good exposition of the Taggart method, but comparatively little of other methods since developed which are of much value and significance. The chapter on Pyorrhea Alveolaris by the editor is the best statement of the history and etiology of this disease we have ever seen in print and although we cannot fully endorse or be entirely satisfied with it we are very glad to have so clear and up-to-date a statement in such accessible form. The technical treatment is not so complete as it should have been made, to meet the needs of students and practitioners who know little of this work. The work of Dr. Prinz on Local Anesthetics is well done, but the general anesthetics, chloroform, ether, somnoform and nitrous oxid do not get the consideration their importance deserves. These anesthetics of course are not so much used in operative dentistry as in oral surgery, yet they are often indicated and are not used because the average dentist has no knowledge or skill in using them.

The book is well printed and should most certainly be in the library of not only students in college, but every practitioner would be wiser and more useful if he had a book of this sort to read when he has a few spare minutes, which are too often worse than wasted on the current literature of the day and the daily newspaper. Lea & Febiger of Philadelphia are the publishers.

